# Insight into the Knowledge, Attitude, Practices, and Barriers Concerning Organ Donation Amongst Undergraduate Students of Pakistan

**DOI:** 10.7759/cureus.5517

**Published:** 2019-08-29

**Authors:** Hania Hasan, Arhama Zehra, Lubna Riaz, Ramsha Riaz

**Affiliations:** 1 Miscellaneous, Dow Medical College, Dow University of Health Sciences (DUHS), Karachi, PAK; 2 Medicine, Dow Medical College, Dow University of Health Sciences (DUHS), Karachi, PAK; 3 Forensic Medicine & Toxicology, Dow Medical College, Dow University of Health Sciences (DUHS), Karachi, PAK

**Keywords:** organ donation, undergraduates, knowledge, attitude, pakistan

## Abstract

Background: In Pakistan, thousands are reported dead due to organ failure annually, owing to the huge discrepancy between the number of people waiting for organ donation and the number of organs available. It is imperative that the young generation, the future donor force, comprehends the concept and importance of organ donation. Hence, this study was conducted to assess the knowledge, attitude, and practices regarding organ donation amongst the youth of Pakistan, and to delineate the factors that motivate or demotivate the new generation for organ donation so our future campaigns may be more successful.

Methods: A cross-sectional study was conducted on undergraduate medical and non-medical students from various universities of Karachi, Pakistan. Using convenience sampling, 450 students were sent online, structured, 11-item questionnaires. The analysis was conducted using Statistical Package for Social Sciences (SPSS version 23.0, IBM Corp., Armonk, NY, US), and associations calculated through chi-square tests. A knowledge score was calculated to reflect a participant's familiarity with organ donation.

Results: 88.7% of students were familiar with organ donation; however, only 34.6% were willing to donate, and 0.6% had donated an organ. Belonging to a medical university and female gender were associated with higher knowledge scores. 'To save lives’ (51.7%) was cited by most students as a factor that would motivate them to donate an organ, while ‘Religion’ (27.6%) was chosen as the most popular factor that demotivates them.

Conclusion: Majority of the students had insufficient overall knowledge about organ donation, highlighting the need for inculcating this topic in the curriculum. The differences in knowledge between medical and non-medical students were inconsistent with their practices, indicating that future campaigns should focus on eliminating barriers to organ donation to facilitate an accepting attitude and increased practices with respect to this topic.

## Introduction

Organ donation is the process of surgically removing an organ or tissue from one person (the organ donor) and placing it into another person (the recipient) [1]. It remains the treatment of choice for organ failure, resulting in an increase in survival rates. The need for organ transplants around the globe has increased by 200% over the past decade, while the number of organ donors remained relatively constant, creating an enormous difference between the number of organs donated and the recipients waiting for donations [2,3].

The United States (US), a highly developed country that provides advanced health care facilities and supports an extensive network for organ donations and transplants, ensures that donors are documented and directed through proper channels. Relatively well-regulated procedures and high availability favor both organ donation and transplantation. However, as of July 2010, 90,000 patients were waiting for a kidney transplant and 116,705 patients waiting for all organs, including liver, heart, lung, pancreas, etc. [3]. As per the data collected by United Network for Organ Sharing (UNOS), more than 6500 people died in 2017 while being on the waitlist or within 30 days of leaving the list, without receiving an organ transplant [4]. Moreover, the most recent data of July 4, 2019, reflects that 113,432 candidates are currently on the US waiting list for organ donation [5].

In Pakistan, on the other hand, the lack of a proper system and an unwillingness found amongst people for organ donation is leading to an increasingly higher demand for organ donation. Around 50,000 people are reported dead due to organ failure each year [6]. With a lack of kidney transplants and liver transplants being conducted in Pakistan, annually, 18,000 people end up with renal failure and 10,000, die of liver failure [7,8]. This grave situation in Pakistan called for an ordinance in the year 2007, allowing cadavers to be used as transferring organs [9]. Furthermore, the Transplantation Society of Pakistan (TSP) was also constructed to promote deceased donors by conducting seminars and meetings to address different aspects of organ donations [10]. However, despite these measures, the knowledge of organ transplantation amongst the Pakistani population was calculated to be 65.5% [11].

This data signifies the need to identify and eliminate barriers in organ donations and devise effective strategies to promote the concept of organ donation. There is a dearth of information on this subject in Pakistan with only one study having been conducted to gain insight into the knowledge, attitude, and practice regarding organ donation amongst the patients coming to the outpatient units of a tertiary care hospital in Karachi, which reported that 59.9% of the people surveyed were willing to donate their organs [12].

Therefore, taking the lack of data regarding this topic into consideration, and the need of promoting awareness in this aspect, our study aimed at assessing the knowledge, attitude, and practice amongst the youth of Pakistan that will form the future donor force of the country. We also aim to delineate the factors that motivate or dissuade Pakistani youth from organ donation, so our future campaigns may be more effective and goal-oriented.

## Materials and methods

This descriptive, cross-sectional study was conducted by employing undergraduate students belonging to various universities of Karachi, Pakistan. The sample population of students was divided into those belonging to the field of medicine, and the rest were categorized as non-medical students. Based on the knowledge, attitude, and practice of organ donation of a previous study, a sample size of 323 was calculated via openepi.com, using 70% anticipated frequency, at a confidence level of 95% and confidence limit of 5% [13].

After a thorough literature search, a structured, self-administered questionnaire was designed based on studies most representative of our topic and was made online through Google forms [14,15]. This study was pilot tested on 15 students, and any errors found were eliminated. The questionnaire was then sent to around 450 students through various social media forums. With the response rate of 82%, 369 of these were returned, and after discarding 16 incomplete forms, we were left with 353 completed forms.

Informed consent was obtained from all participants, and confidentiality and anonymity were maintained by not asking for names in the online form. The questionnaire included a total of 11 questions, beginning with the respondent’s demographic information, followed by questions that accompanied ‘yes/no/don't know’ options; assessing knowledge, attitude, and practice amongst these students (see Appendices). The questions inquired about the responder's familiarity with organ donation and donators. It then assessed their knowledge by asking if they knew about organ viability, age limit, procedure, organ donation card, ‘transplantation of human organs and tissue bill 2007’, and brain death. The attitude was assessed by asking if they would be willing to donate organs, followed by factors that motivate or demotivate them to donate organs. They were also asked who they would be willing to donate their organs to and if they find it ethical to donate organs for money. Questions on practice included donating an organ and signing the organ donation card.

Data were entered into Microsoft Excel 2016 and analyzed using IBM Statistical Package for the Social Sciences (SPSS) 23.0 (IBM Corp., Armonk, New York). Descriptive statistical analysis, including frequency, percentages, and chi-square test to derive P values were used to characterize and analyze the data. A P value <0.05 was considered statistically significant. Out of the seven questions asked regarding students’ knowledge of organ donation, a mean knowledge score was calculated. Those achieving >/= 50% were regarded as having 'Above Average Knowledge', while those scoring < 50% were regarded as having 'Below Average Knowledge' [12].

## Results

Our study comprised of a total of 353 undergraduate students, the majority of whom were females. The study participants from medical universities and non-medical universities were roughly equal in distribution (44% and 56% respectively). There was no significant difference in the mean ages of both the groups (P=0.58). The demographics are summarised in Table 1.

**Table 1:** General demographics of study participants (n=353) *P value calculated using chi-square for categorical variables and t test for continuous variable. SD: Standard Deviation

Field of education (%)	Medical Students	Non-medical Students	P value*
Gender			
Male	26 (7.4)	112 (31.7)	0.00
Female	131 (37.1)	84 (23.7)	
Mean age (years) ± SD	20.6 ± 1.6	20.5 ± 1.4	0.58

**Figure 1:**
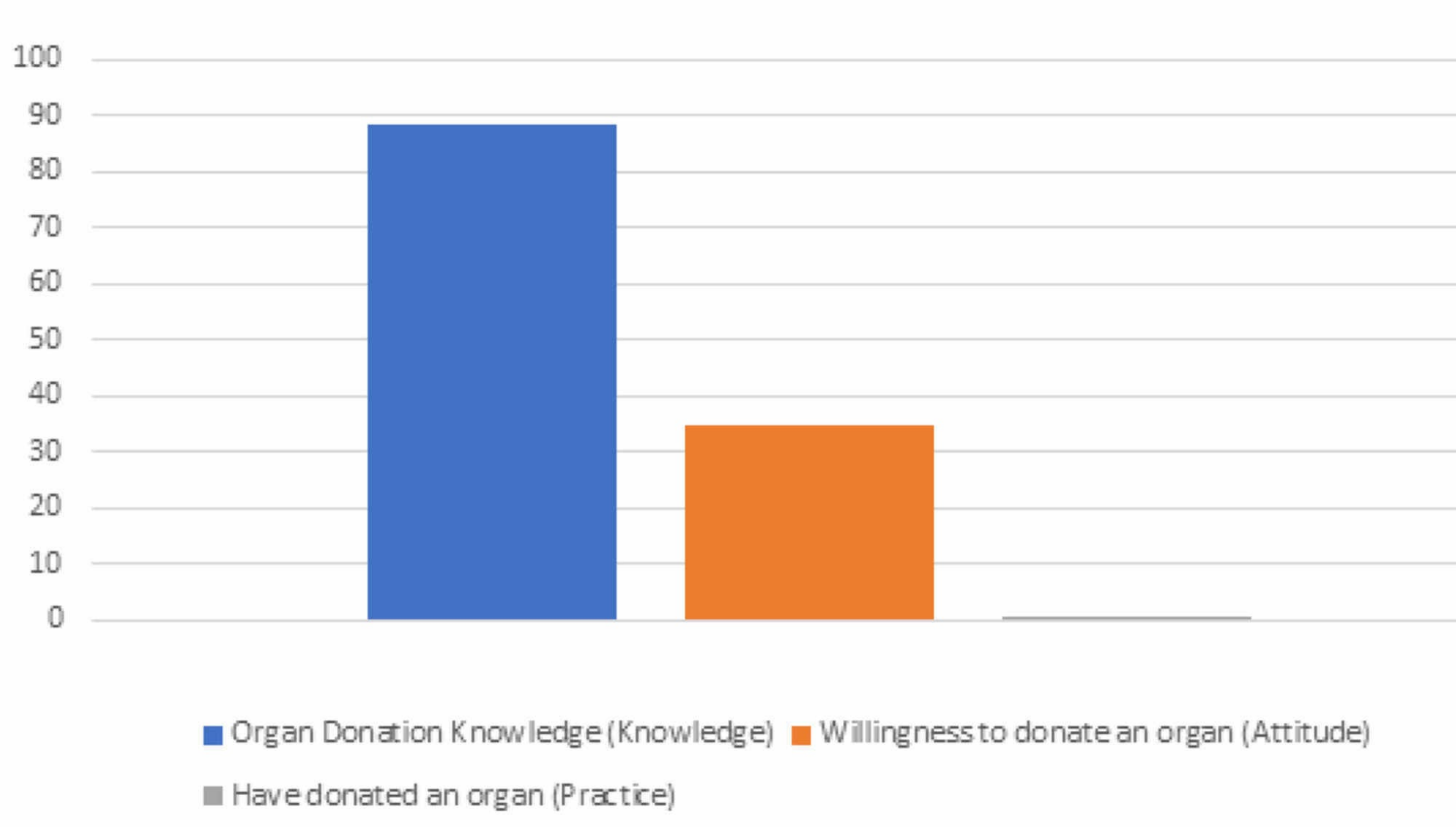
Comparison of knowledge, attitude, and practice towards organ donation

Most students had heard about organ donation (88.7%); however, very few were willing to donate or had donated an organ (34.6% and 0.6%, respectively). This comparison is illustrated in Figure 1.

Table 2 demonstrates the association between socio-demographic characteristics and knowledge of respondents. Students belonging to the medical field had a higher knowledge score than students from a non-medical background (P=0.00). Furthermore, it was also observed that female gender was associated with a higher knowledge score when compared to males (P=0.01).

**Table 2:** Mean knowledge scores with respect to gender and field of study * P value calculated using chi square test

		Below Average Score	Above Average Score	P value*
		N (%)	N (%)	
Field of Education (N)	Medical (157)	55 (35.0)	102 (65)	0.00
	Non-medical (196)	127 (64.8)	69 (35.2)	
Gender (N)	Female (215)	99 (46)	116 (54)	0.01
	Male (138)	83 (60.1)	55 (39.9)	

The knowledge, attitude, and practices towards organ donation are illustrated in Table 3. Majority of people in both groups had heard about organ donation (96.1%, 82.7%). There was a statistically significant difference in five out of seven questions assessing ‘Knowledge’ between the two groups (P<0.01). Willingness to donate an organ and to sign a donation card was higher amongst medical students, and this difference was also statistically significant (P=0.02 and 0.00, respectively). However, in both groups, the percentage for these variables remained less than half of the study population (41.4% and 29.1%, 42% and 24% respectively). With regards to the ethical aspect of organ donation, only a minority thought that it was ethical to sell organs for money. When asked “Who would you prefer donating your organ to?”, the majority of the participants had no preference for donation (47%). Furthermore, only a few participants had ever donated an organ or signed an organ donation card (1.3% and 5.2% respectively).

**Table 3:** Comparison of knowledge, attitude, and practices amongst medical and non-medical students *Calculated using chi square test

	Medical (%)	Non-medical (%)	P value*
Knowledge (‘yes’ responses)			
Are you familiar with organ donation?	151 (96.1)	162 (82.7)	0.00
Do you know of anybody who has donated an organ?	42 (26.8)	44 (22.4)	0.349
Is there a time duration for which organ remains viable for transplant?	136 (86.6)	116 (59.2)	0.00
Is there is an age limit for organ donation?	84 (53.5)	73 (37.2)	0.00
Do you have any knowledge regarding the procedure of organ transplantation?	47 (29.9)	31 (15.8)	0.00
Do you know about organ donation card?	59 (37.6)	50 (25.5)	0.02
Do you know about the ‘transplantation of human organs and tissues bill, 2007’?	8 (5.10)	12 (6.1)	0.68
Attitude (‘yes’ responses)			
Do you think it is okay to donate the organ of a comatose person without his/her consent?	5 (3.2)	7 (3.6)	0.13
Would you be willing to donate an organ?	65 (41.4)	57 (29.1)	0.02
Would you be willing to sign an organ donation card	66 (42.0)	47 (24.0)	0.00
Do you think it is ethical to sell organs for money?	11 (7.0)	21 (10.7)	0.50
Practice (‘yes’ responses)			
Have you ever donated an organ?	2 (1.3)	0 (0.0)	0.11
Have you signed an organ donation card?	5 (3.2)	4 (2.0)	0.50

**Figure 2:**
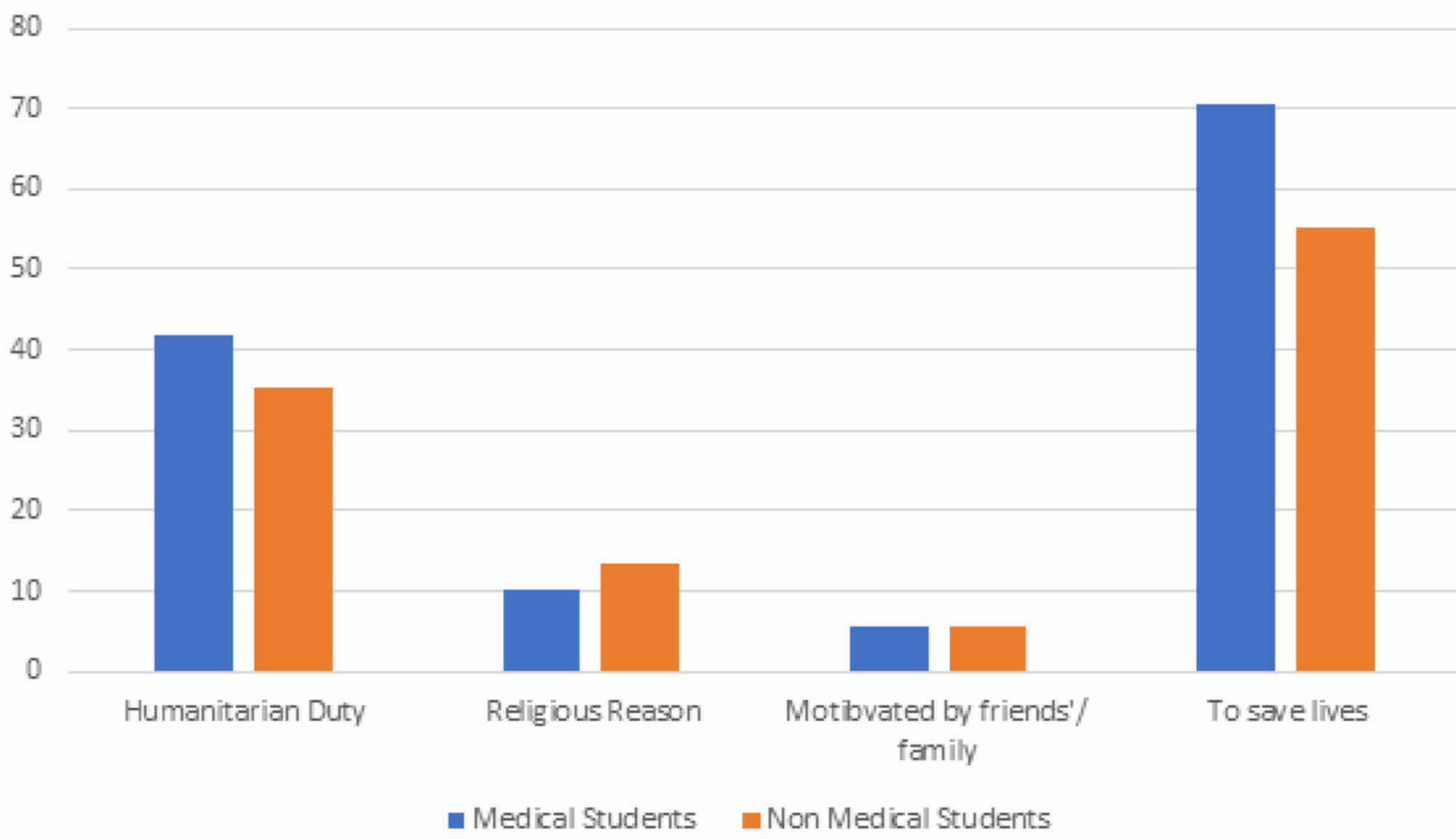
Reasons cited for donating organs

**Figure 3:**
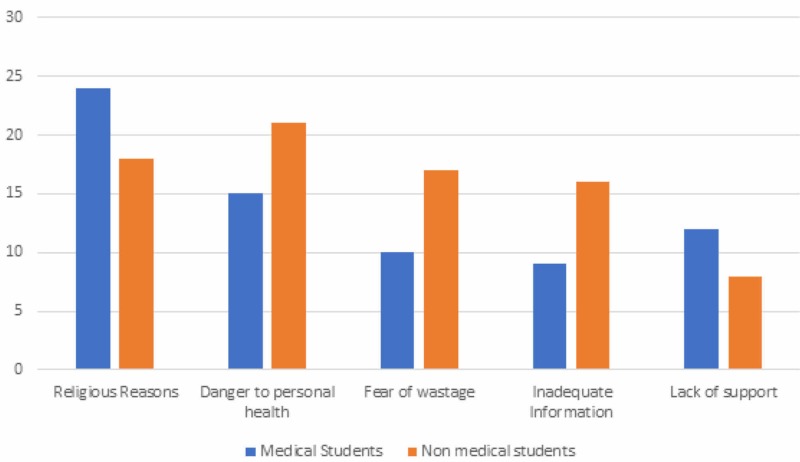
Reasons cited for not donating organs

When asked about possible reasons for donating their organs, the majority of the people chose “To save lives” (51.7%) followed by “Humanitarian duty” (33.7%). In a separate question, they were also asked the possible reasons for not donating their organs, 27.6% of students chose “Religion”, followed by “Risk to personal health” (24.9%). Factors chosen as “Others” included “mishandling of organs”, “personal health concerns” and “it is a distasteful thing”. Majority of non-medical students had concerns regarding fear of wastage of organs and dangers to personal health as compared to medical ones. These reasons and obstacles are summarized in Figures 2, 3.

## Discussion

Considering the wide mismatch between the demand for organ transplantation and its supply, it is imperative that the young generation-the future donor force-comprehends the concept of organ donation and eliminates all misconceptions regarding it. Thus, we aimed to assess the knowledge, attitude, and practices of undergraduate students regarding organ donation and explore the factors that motivate them and those that prevent them from donating organs.

Overall, knowledge of undergraduates regarding organ donation was lower (48%) as compared to a study conducted previously in Pakistan on the adult population (59.9%) [12]. This contrast can be attributed to the fact that adults, as compared to youngsters, have more exposure and knowledge regarding social issues and more experience to be motivated to save a life. They may also be mature enough to value someone’s life since the younger population is more driven to enjoy their youth and therefore, may be less keen unless they have a personal/family experience. However, this low percentage emphasizes the need of inculcating knowledge amongst undergraduate students, since this topic is not stressed enough in our educational system. This also calls for more awareness for the youth of the country by conducting mass campaigns through radio, TV, billboards, especially social media as it has a vital role in shaping the minds of this generation and is one of the most effective ways to reach our target population. Another possible way to increase awareness is to include well-known personalities (e.g., actors, models, cricketers) which youth looks up to, to spread this message through advertisements and campaigns. There is also a need to highlight the importance of donation to students by conveying statistics on the shortage of organs and the number of lives that can potentially be saved by their donation. Such sessions should also include videos recorded with transplant patients, donors, and their families to inspire them even further.

Our study also found out that knowledge regarding organ donation was higher amongst medical students as compared to non-medical students (65% and 35%, respectively). This is similar to studies done in Greece and the UK, in which a similar pattern was observed [14,16]. More medical students were also willing to donate as compared to non-medical students (41.4% vs. 29.1%). This is likely due to the impact medical education has on a person’s attitude, and greater exposure of medical students to organ failure patients and awareness of such topics in a hospital environment [16]. Our study showed little knowledge (5.7%) regarding “Transplantation of Human Tissues and Organ Bill 2007” as compared to a similar study previously conducted in Pakistan amongst medical students (9%). This decreasing trend may be due to the difficulty in understanding the legal terms amongst undergraduate students and hence, once again emphasizes the need of incorporating this topic in the curricula, and spreading awareness through media and technology, so that individuals understand their responsibility in this regard [16].

In our study, more than half of females and less than half of males had adequate knowledge of the subject, and this difference was statistically significant. This is in contrast to the study done on an urban Indian adult population where a greater number of males were found to have adequate knowledge [17]. This difference could be due to the difference in the study sample since in our study, majority of females were from medical universities and are expected to have more knowledge on the subject as compared to males, which were majorly from non-medical universities. It could also be due to cultural differences between the two countries.

Our study also revealed that 65% of the sample population was not willing to donate their organs. These alarming statistics demonstrate the need for immediate intervention to modify the attitude of students. The discrepancy between having adequate knowledge (48%) and willingness to donate (35%) shows that participation in organ donation has not reached the required level, which could be due to insufficient emphasis, lack of exposure, lack of understanding about the process, fear of commercial usage of organs, lack of counselling, and lack of campaigns aiming at masses, along with the lack of focus on addressing potential donor issues [18-21].

More medical students were willing to sign an organ donation card and to donate an organ as compared to their non-medical counterparts. This significant finding reflects that the fact that medical students were more knowledgeable on the subject as demonstrated in our results and that they are also more empathetic towards people because of exposure they get in their medical rotations and understand the need of a person who is dependent on someone for his survival.

The fact that difference in knowledge and attitude was significant between two specialties but the practice was not, shows that at the end of the day, it does not matter whether students were from medical specialty or not since having more knowledge or a positive attitude has not directly affected their practice. Thus, there is a need to focus on youth in general, encourage them to donate organs, and concentrate on potential obstacles they face in the process.

We also found out that the majority of students had no preference for donation and could donate organs to whoever needs them. This gives us an idea that partiality won’t be an issue if we manage to encourage students to donate organs since most students will donate regardless of gender, caste, status, etc. 

Majority of students stated “Religion” as a reason for not donating their organs. There was a statistically significant association between willingness to donate and religious reason for not donating organs (p=0.047). This view is supported by a study done in Saudi Arabia, which concluded that clearing Islamic misconceptions amongst people would have the strongest positive influence on the willingness to donate [22]. This, however, is in contrast to a study in India in which religious reasons were the least common factors for the reluctant attitude towards organ donation that is probably due to a difference in the religion of the majority population [13]. The second most popular reason cited was “Danger to personal health” which is similar to a study conducted in Saudi Arabia on university students, where “fear of side effects” was the major reason for the refusal to donate organs [23]. These statistics reflect a general lack of understanding amongst undergraduates on this topic and calls for counseling sessions to be conducted in universities, which explain the process and remove students' misconceptions. Factual knowledge specifically needs to be inculcated, and issues should be addressed to eliminate this mistaken belief.

When asked about the motivation behind organ donation, most people elected “to save someone’s life”. This is consistent with a study done previously in Pakistan on the adult population, suggesting that people, in general, are driven by noble purposes [12]. However, the fact that some also chose “for money” as a reason blurs the boundary between right and wrong due to excessive organ trading in the country. This calls for effective strategies to not only control organ trading but also to counsel students about the legal and ethical issues concerning organ donation and transplantation.

While our study addresses important issues, it has certain limitations. Firstly, we employed convenience sampling in our study, and our sample was not randomized and not matched based on age and gender. Moreover, our sample size was small and did not include undergraduates of all universities in Karachi; thus, these results might not be an accurate representation of all students. We assessed students’ knowledge based on seven questions, which could lead to an inaccurate interpretation of their overall knowledge of the subject.

## Conclusions

In our study, overall knowledge amongst undergraduates regarding organ donation is inadequate, and attitude and actions are inconsistent with one another. The field-of-study had a significant association with both knowledge and attitude. The nobility of the act was a major influencer, while religion was a major hindrance to organ donation. Hence, we propose conducting large-scale studies in colleges/universities of Pakistan, aiming for a more detailed assessment of the knowledge of organ donation to counsel and educate students more effectively.

## Appendices

Questionnaire:

Age: _____

Gender a) Male b) Female 

Specialty of study a) medical b) non-medical 

Religion a) Muslim b) Non-Muslim  

Are you familiar with organ donation? a) yes b) no 

Does your religion allow you to donate organs? a) yes b) no c) don’t know 

Do you know of anybody who has donated an organ? a) yes b) no 

 Have you donated an organ? a) yes b) no 

 If no, would you be willing to donate an organ in future? a) yes b) no c) may be 

 If yes, why? (you may choose more than one) a) humanitarian duty) b) to save lives c) religious concerns d) motivated by family or friends e) other 

Who would you like to donate your organ to? 

A) Family 

b) Stranger 

c) Colleague/ friend 

 If no, why? (you may select more than one) a) inadequate information b)danger issues c)religious reasons d)lack of family and relatives support 

e) Fear that organs could be wasted/mistreated f) other  

Is there a time duration for which organ remains viable for transplant? a) yes b) no c) don’t know 
 

Is there is an age limit for organ donation? a) yes b) no c) don’t know 

Do you have any knowledge regarding the procedure of organ transplantation? a) yes b) no 

Do you know about organ donation card? a) yes b) no c) only heard the name 

Have you signed a donation card? a) Yes b) no 

  If no, will you be willing to sign a donation card? a) Yes b) no c) not sure  

Do you have the concept of brain death? a) yes b) no c) slightly 
 
 

Do you know about the ‘transplantation of human organs and tissues bill 2007’ ? a) yes b) no 

Do you think it is ethical to donate an organ for money? a) yes b) no 
 
